# NLRP3 promotes the proliferation and metastasis of colorectal cancer by regulating the S6K1-GLI1 signaling pathway

**DOI:** 10.7150/jca.101285

**Published:** 2025-01-01

**Authors:** Si Li, Xuchao Wang, Li Hong, Zirui Zhuang, Pei Yang, Yu Chen, Yizhou Yao, Linhua Jiang, Xinguo Zhu, Bin Wang

**Affiliations:** Department of General Surgery, The First Affiliated Hospital of Soochow University, Suzhou, Jiangsu, China.

**Keywords:** CRC, NLRP3, EMT process, S6K1-GLI1 pathway, Proliferation, and metastasis.

## Abstract

**Background:** Colorectal cancer (CRC) is the primary cause of cancer-related mortality globally. Research indicates that CRC is associated with the dysregulation of NLRP3 expression. Therefore, further investigation is warranted into the correlation between NLRP3 and CRC proliferation and metastasis.

**Methods:** NLRP3 and GLI1 expression levels were assessed in tumor tissues using qPCR and bioinformatics analysis. We performed Western blot, CCK8 assay, Colony formation assay, Transwell assay, and mouse xenografts to investigate the effects of NLRP3 on the proliferation and migration of CRC cells while identifying the potential underlying mechanisms involved.

**Results:** Our research demonstrated that elevated NLRP3 levels in CRC tissue correlated with adverse patient outcomes. Enhanced NLRP3 expression significantly affects progression-free and relapse-free survival. Furthermore, suppressing NLRP3 expression effectively inhibited the proliferation and migration of CRC cells while impeding epithelial-mesenchymal transition (EMT) signaling and the S6K1-GLI1 pathway. Notably, the mouse xenotransplantation study validated that deleting NLRP3 suppresses CRC development.

**Conclusion:** NLRP3 facilitates CRC progression via the EMT and the S6K1-GLI1 signaling pathway, providing a promising target against CRC patients.

## Introduction

In recent years, CRC occurrence has been consistently increasing, making it the third most frequently diagnosed malignant tumor worldwide and ranking fourth as a reason behind cancer-related mortality 1, 2. Despite significant advancements in surgical techniques, targeted therapy, and immunotherapy, which have notably improved the overall survival rates of CRC patients, the survival prognosis remains poor because of the high number of patients presenting at advanced or metastatic stages 1, 3. Therefore, investigating the mechanisms involving the proliferation, invasion, and metastasis of CRC remains crucial in identifying effective targets to inhibit tumor progression.

The NLRP3 inflammasome is a cytoplasmic protein complex comprising the NLRP3 receptor, apoptosis-associated speck-like protein (ASC), and a precursor cysteine aspartic protease (pro-Caspase1). It is a crucial complex associated with the innate immune response. Activating NLRP3 bodies can facilitate the proliferation and metastasis of lung cancer A549 cells 4, 5. The NLRP3 inflammasome in gastric cancer promotes cell differentiation by participating in cyclin-D1 while producing IL-1β. IL-1β binds to its receptor and activates the NF-kB pathway, leading to JNK signaling, which induces tumor proliferation, invasion, and cancer development 6. Furthermore, the NLRP3 inflammasome is crucial in maintaining intestinal homeostasis and regulating immune responses. However, its specific involvement and the underlying mechanism in regulating CRC remains unelucidated 7, 8.

Epithelial-mesenchymal transition (EMT) is the process by which epithelial cells, under specific conditions, lose their original polarity and tight junction characteristics, gradually transforming into cells possessing mesenchymal features. This is accompanied by reduced epithelial cell markers like E-cadherin and increased mesenchymal cell markers like N-cadherin and Vimentin. The EMT process imbibes cancer cells with improved invasiveness and migratory capabilities. Studies have indicated that activating the NLRP3 inflammasome can regulate EMT-related gene expression via complex molecular mechanisms 9, 10. This potentially plays a crucial role in promoting invasion and CRC cell metastasis.

The Hedgehog signaling pathway is highly conserved in evolution, and its aberrant activation is closely linked with the proliferation, invasion, migration, and drug resistance of various tumor cell types 11-13. In the Hedgehog signaling pathway, other than the canonical PTCH-SMO-GLI regulatory axis, GLI1 protein expression is modulated by different non-SMO-dependent pathways, including the PI3K-Akt, MAPK/ERK, and KRAS pathways 14, 15.

NLRP3 activates the tumor-associated PI3K-AKT signaling pathway 4, 16, 17. S6K1 is the critical effector of the mTOR signaling pathway and is fundamental in triggering and driving CRC development. Therefore, exploring the regulatory interactions between NLRP3 and the S6K1-GLI1 pathway in CRC can help elucidate the contributions of NLRP3 to the tumor development process. This study observed a substantial NLRP3 upregulation in CRC tissues, which correlated strongly with disease progression and poor prognosis. Our *in vitro* and *in vivo* experiments investigated the impact of NLRP3 on the proliferation and migration of CRC tumors and its potential correlation with the EMT process and the S6K1-GLI1 signaling pathway, providing novel insights for targeted CRC therapy.

## Materials and methods

### Human CRC tissues

CRC tissues were recruited from 27 patients at the First Affiliated Hospital of Soochow University in 2019. All the patients had undergone complete radical surgery, and subsequent histopathological examination established the CRC diagnosis. The Independent Ethics Committee of the First Affiliated Hospital of Soochow University (2020076) approved the study.

### Immunohistochemistry

Subcutaneous tumor tissues were embedded in paraffin, cut into 3 μm sections, and subjected to IHC 18. The sections were incubated using anti-NLRP3 (ABclonal, China) and anti-p-AKT, p-S6K1, GLI1, SMO (CST, USA) at a 1:100 dilution overnight at 4℃. A tissue staining kit (Zhongshan Biotechnology, China) helped visualize tissue expression. The IHC score was determined using the intensity multiple (0, negative; 1, weak; 2, moderate; 3, strong) and extent (0, <5%; 1, 5-25%; 2, 25-50%; 3,50-75%; 4, >75%) scores 19.

### Cell culture

SW480, LOVO, and HCT116 cells were grown in RPMI 1640 medium (HyClone, USA) with 10% FBS (Gibco, USA). RKO and NCM460 cells were cultured in DMEM medium (HyClone, USA) and supplemented with 10% FBS.

### Transfection of shRNA

Lentiviral transfection occurred when cell growth and fusion reached approximately 70%. The requisite lentivirus was incorporated into the cell culture medium based on the instructions (shRNA and negative control lentivirus were designed and synthesized by Suzhou Jima Gene Co., LTD.). After 48 hours of virus transfection, stable cell lines were screened by adding a medium containing puromycin at 2 μg/ml. Subsequently, the medium was replaced using puromycin medium at 12 hours, repeating the process 2-3 times.

### RNA extraction and qRT-PCR

Total RNA was extracted from the cells using TRIzol Reagent (Takara, Japan), and the concentration and purity of the extracted RNA were verified using a NanoDrop 2000 spectrophotometer. The qRT-PCR was performed with the TB Green Premix Ex Taq II kit (Takara, Japan) using the real-time PCR system. The relative expression levels of target genes were determined using the 2^-ΔΔCT^ method, with GAPDH becoming the internal control for normalization.

### Western blotting

After Electrophoretic separation, Transmembrane purification, and Blockade, the protein was incubated at 4℃ overnight using a primary antibody. This was followed by treatment with secondary antibodies. The ImageJ software helped evaluate the quality of the Western blot images.

### Cell viability assay

The cells were cultured for days 1, 2, 3, 4, and 5, followed by the addition of CCK-8 reagent (Vazyme Biotech Co., Ltd, China). Subsequently, the absorbance at OD450nm was measured with a microplate reader, and the experiment was repeated at least three times.

### Colony formation assay

The cell suspension was transferred into a new 6-well plate. After culturing in the medium for two weeks, the cells were stained using crystal violet. Subsequently, the number of colonies formed was determined using a microscope. The experiment was repeated at least three times.

### Transwell assay

The Transwell chamber was kept in a 12-well plate, and 200µL of cell suspension was added to the upper chamber. Subsequently, 700µL of complete medium was added to the lower chamber. After 24 hours, the cells were stained using crystal violet, imaged with an inverted fluorescence microscope, and quantified. The experiment was repeated at least three times.

### Mouse xenografts

This study used 4-week-old specific pathogen-free (SPF) BALB/c-nu nude mice as experimental animals sourced from Shanghai Slack Laboratory Animal Co., LTD. After a one-week acclimatization period, the mice were randomly allocated into the NLRP3-knockdown and negative control groups. On day 0, 1×10^7^ SW480 cells were subcutaneously injected into the right dorsal side of each mouse. Once the tumors became palpable at the injection site, the mice started a triweekly weight and tumor volume measurement regimen. The short diameter a (mm) and long diameter b (mm) of the subcutaneous tumor in nude mice were determined using vernier calipers. The subcutaneous tumor volume was calculated as volume (mm³) = (a² × b) / 2. After 28 days of monitoring, the nude mice were humanely euthanized. Then, their subcutaneous tumors were removed for weight analysis and documentation. All experimental procedures were approved by the Animal Ethics Committee of the First Affiliated Hospital of Soochow University (2020076).

### Statistical analysis

The statistical analysis was conducted using SPSS 22.0. The data were represented as mean ± SD. The two groups were compared using the Student's T-test, while ANOVA (Analysis of Variance) helped compare multiple groups. The plots were generated using GraphPad Prism 8 and R (version 3.6.1, http://cran.r-project.org/), with *P* < 0.05 being statistically significant.

## Results

### NLRP3 is highly expressed in CRC tissues and predicts poor survival

**Figure 1:**
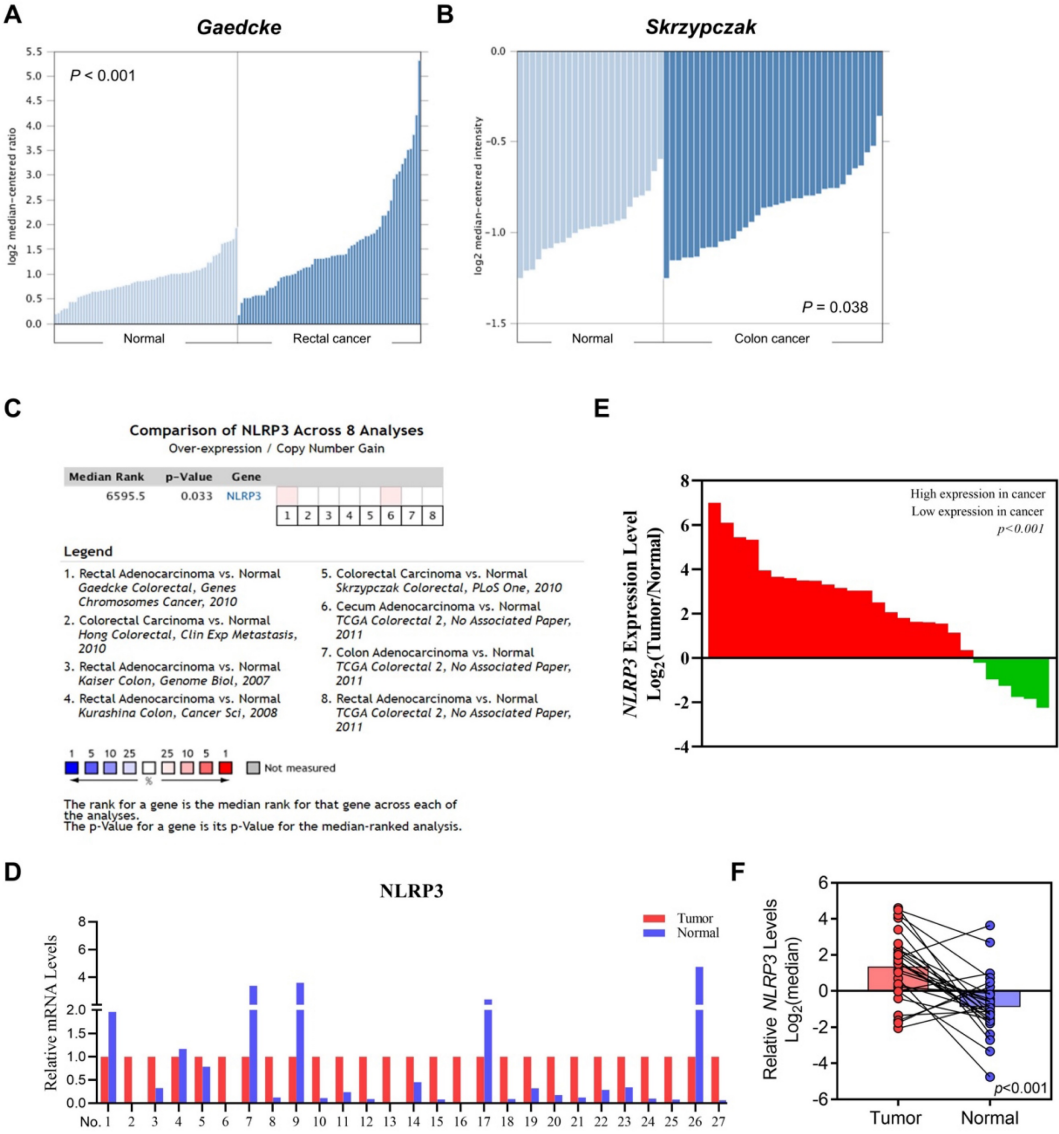
**The differential NLRP3 expression in intestinal cancer and normal intestinal tissues.** (A-B) Evaluating NLRP3 expression in colorectal cancer and normal tissues with the Gaedcke (A) and Skrzypczak (B) datasets. (C) The comprehensive analysis of NLRP3 expression levels in CRC tissues across datasets. (D) NLRP3 expression levels in tumor tissues were standardized to 1 within each tissue pair. Moreover, its expression level in adjacent normal tissues was assessed. (E) Array data based on the ratio of tumor to normal tissues, from highest to lowest. (F) Log2 index pairing of the qPCR results, normalizing the NLRP3 expression level in tumor tissues in the first tissue pair to an index of 0.

We used Oncomine to analyze its differential expression between tumor samples and corresponding healthy controls to investigate the role of NLRP3 in CRC pathogenesis. Notably, in both Gaedcke's (rectal cancers) and Skrzypczak's (colon cancers) datasets, substantial upregulations were observed relative to their respective normal counterparts (Figures 1A and 1B). This trend persisted across eight curated datasets within Oncomine, validating heightened levels of colonic malignancies (Figure 1C). Furthermore, our exploration of patient outcomes indicated an association between elevated NLPR3 expression and progression-free and recurrence-free survival. This was evidenced by analyses of the GSE17536 and GSE103479 datasets (Figures S1A and S1B). Finally, qRT‒PCR assays of matched tumor-normal pairs from 27 patients confirmed elevated expression in malignant specimens compared to their noncancerous counterparts (Figures 1D‒F).

### The association between the S6K1-GLI1 pathway and NLRP3 expression in CRC

**Figure 2:**
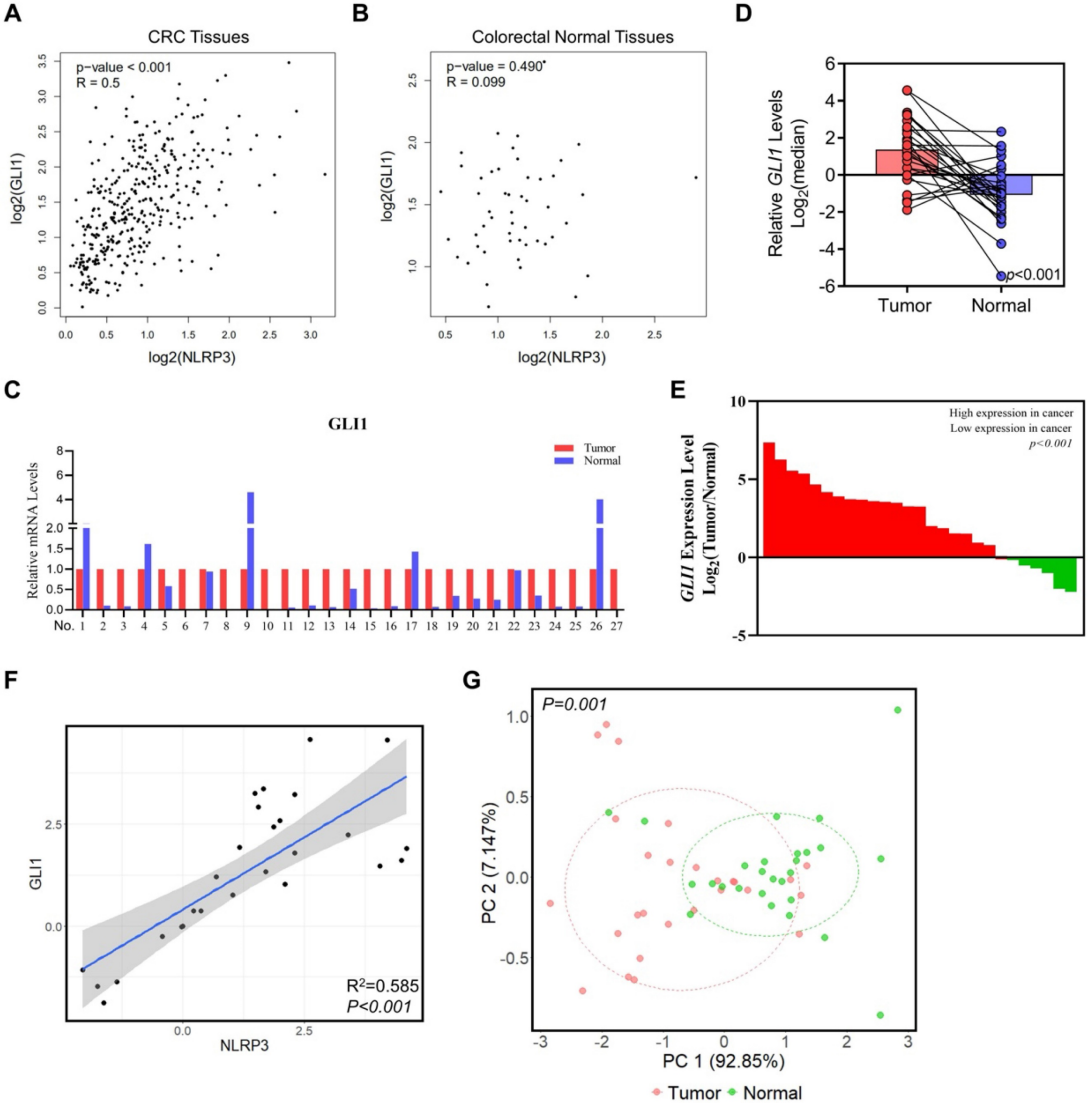
**The relationships between the NLRP3 and GLI1 expression levels in CRC.** (A, B) The correlation between NLRP3 and GLI1 expression levels in CRC and normal colorectal tissues within the TCGA database was analyzed using the GEPIA platform. (C) GLI1 expression level in tumor tissues was standardized to 1 within each tissue pair. Moreover, its expression level in adjacent normal tissues was determined. (D) Log2 index pairing analysis of the qPCR results, normalizing the GLI1 expression level in tumor tissues in the first tissue pair to an index of 0. (E) Array data based on the ratio of tumor to normal tissues, from highest to lowest. (F) Linear correlation analysis of NLRP3 and GLI1 mRNA expression. (G) The clustering analysis was performed on the mRNA expression levels of GLI1 and NLRP3 within cancer and adjacent normal tissues.

Studies have demonstrated a robust correlation between the expression of GLI1 and p-S6K1 in malignant tumors, particularly among patients having advanced-stage tumors. Additionally, our prior studies have revealed a direct positive relationship between the expression of NLRP3 and S6K1 20. Subsequently, we analyzed NLRP3 and GLI1 expression in CRC and normal tissues from the TCGA database using the GEPIA platform. The findings demonstrated a positive correlation between NLRP3 and GLI1 expression in CRC tissues (Figure 2A). NLRP3 and GLI1 expression in normal colorectal tissues did not correlate significantly (Figure 2B).

In support of our aforementioned perspectives, 27 pairs of CRC tissue samples and their corresponding adjacent normal tissue samples were collected for analysis using qRT-PCR and assessing GLI1 expression levels. Our findings demonstrated a significant decline in GLI1 expression in matched normal tissue compared to intestinal cancer tissue (Figures 2C-E). Furthermore, linear correlation analysis showed a positive correlation between the mRNA expression of NLRP3 and GLI1 in bowel cancer tissue (Figure 2F). Subsequent cluster analysis based on mRNA expression indicated effective differentiation between tumor and normal tissues along the PC1 axis depending on their respective GLI1 and NLRP3 levels (Figure 2G). Significantly, a distinct disparity was observed in the expression of NLRP3 and GLI1 between the two groups.

### NLRP3 enhances the proliferation and migration ability of CRC cells

**Figure 3:**
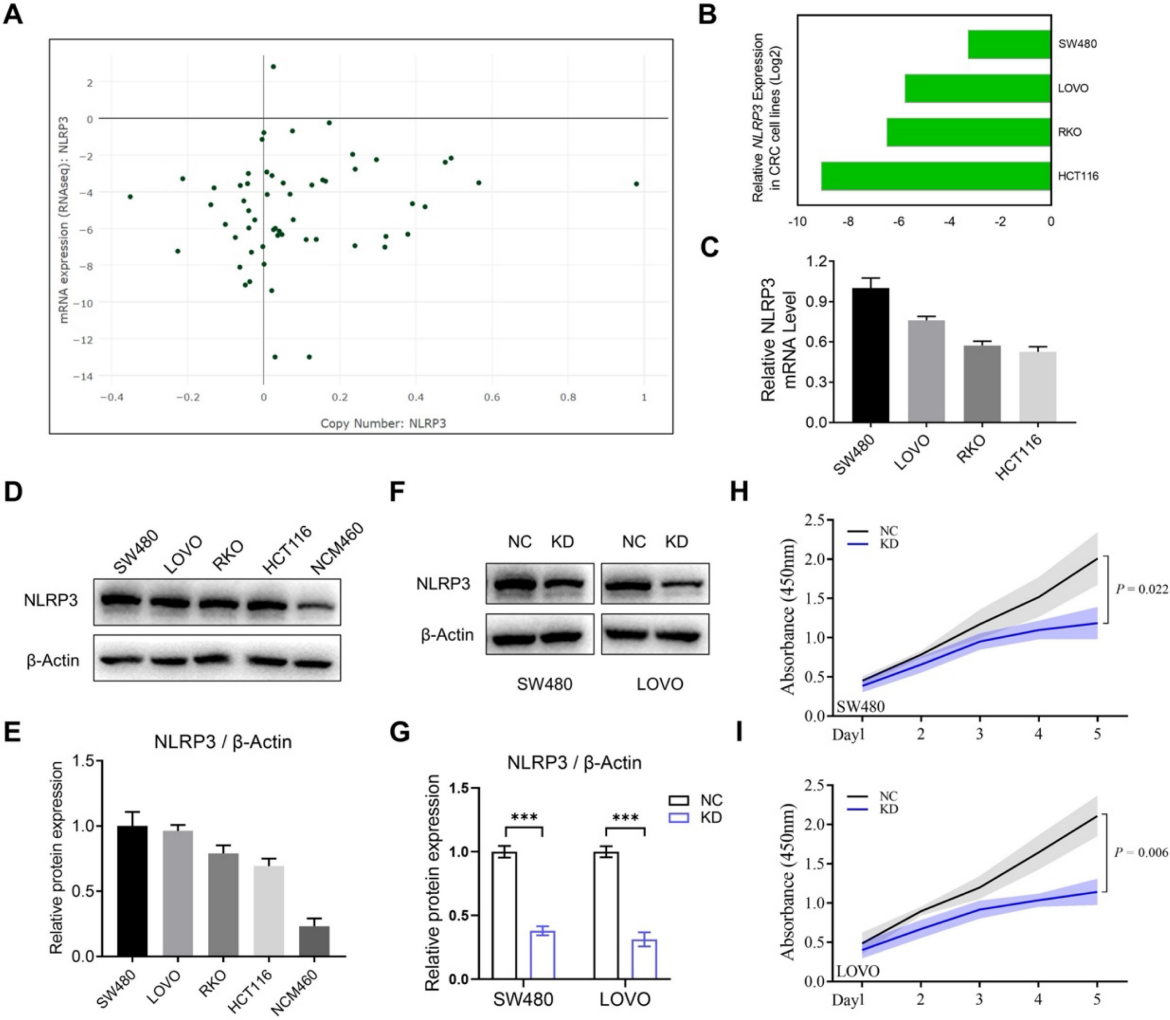
** NLRP3 expression in CRC cell lines was examined and validated.** (A) NLRP3 expression levels in CRC cell lines were analyzed using the CCLE cell database, with green dots representing CRC cell lines. (B) Expression analysis of CRC cell lines within the CCLE database was performed. (C) q-PCR helped detect the mRNA expression levels of NLRP3 in established CRC cell lines. (D, E) Western blotting helped assess the expression of NLRP3 in the CRC cell lines SW480, LOVO, RKO, and HCT116 and the intestinal epithelial cell line NCM460. The ImageJ software helped semi-quantitatively analyze the results. (F, G) The effect of NLRP3 knockdown on CRC cells was evaluated through Western blotting and semi-quantitatively analyzed using the ImageJ software. (H, I) The proliferation capacity of CRC cells within the NC and NLRP3 knockdown groups was assessed with a CCK-8 kit. NC, negative control, control group; KD, NLRP3-knockdown, NLRP3-knockdown group. *** *P* < 0.001.

The study elucidated the effect of NLRP3 expression on the proliferation and migration of CRC cells. Initially, the expression level of NLRP3 in various CRC cell lines was analyzed using the CCLE data platform, indicating enhanced expression of NLRP3 in these cells (Figures 3A and 3B). Subsequently, qPCR validation validated these findings (Figure 3C). Furthermore, Western blotting assessed NLRP3 protein expression levels in CRC and normal intestinal epithelial cell lines. NLRP3 expression was more significant in SW480 and LOVO cells compared to NCM460 normal intestinal epithelial cells. However, no significant differences could be observed among the four CRC cell lines (Figures 3D and 3E).

**Figure 4:**
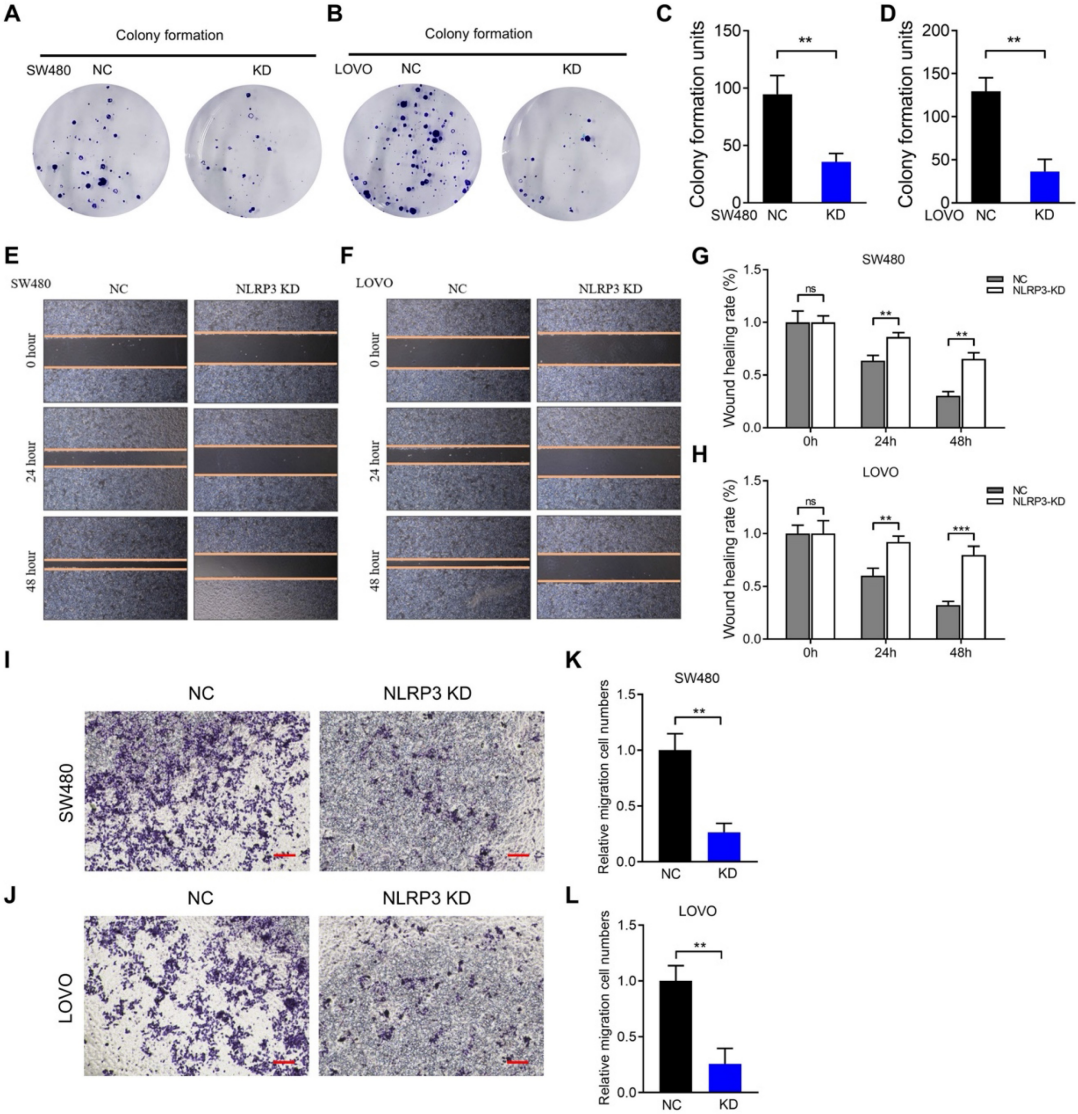
** NLRP3 impact on the growth and migration ability of CRC cells.** (A-D) Cloning experiments were conducted to evaluate the proliferative capacity of the carcinoma cell lines within the NC and NLRP3 knockdown groups, followed by quantitative analysis. (E-H) Scratch assays helped evaluate the migratory potential of the NC and NLRP3 knockdown groups with subsequent quantitative analysis. (I-L) Transwell assays were used to determine the migration ability of the NC and NLRP3 knockdown groups. NC, negative control, control group; KD, NLRP3-knockdown, NLRP3-knockdown group. ns, no significance, ** *P* < 0.01, *** *P* < 0.001.

Based on the results mentioned above, the CRC cell lines SW480 and LOVO were selected for subsequent experiments. Stably transfected NLRP3 knockdown cell lines were established with the NLRP3-specific shRNA transfection technique, and the knockdown effect was evaluated using Western blotting. The findings indicated a significant decline in NLRP3 expression in the knockdown cell lines compared to the control cells (Figures 3F and 3G). Subsequently, CCK-8 and colony formation assays were performed using NLRP3 knockdown CRC cells to evaluate the proliferation of cell lines. The proliferation ability of these cells was significantly suppressed after NLRP3 knockdown (Figures 3H-I, Figures 4A-D). We evaluated its impact on cell migration ability, revealing that reducing NLRP3 expression effectively inhibits bowel cancer cell proliferation. The scratch and Transwell assay results indicated that downregulating NLRP3 expression significantly affected bowel cancer cell migration (Figures 4E-L). These findings suggest a potential role for NLRP3 in enhancing the proliferation and migration of CRC cells. Notably, these processes are substantially inhibited after downregulating NLRP3 expression.

### NLRP3 promotes S6K1-GLI1 signaling and the process of EMT

**Figure 5:**
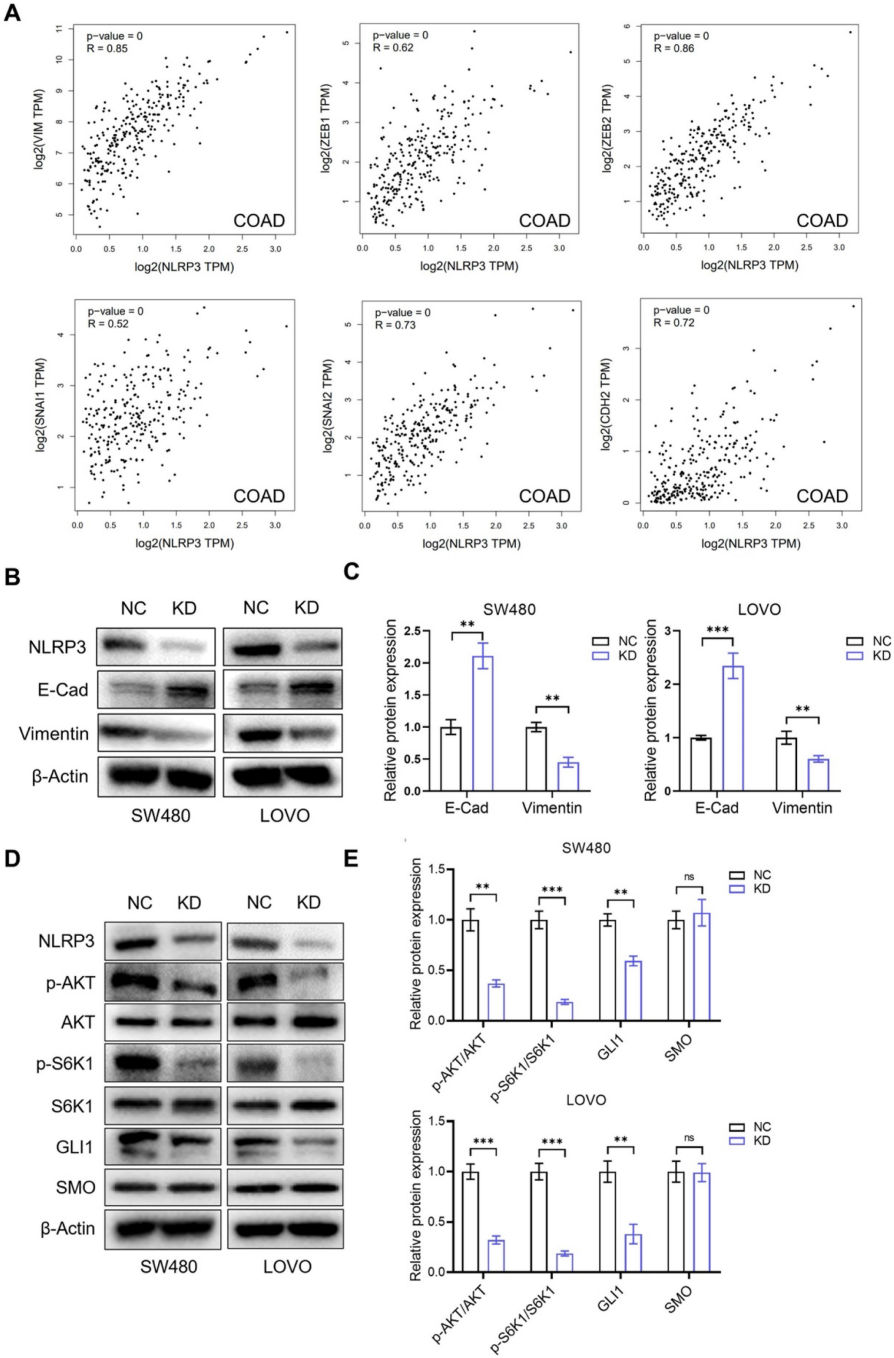
** NLRP3 regulated the impact of S6K1-GLI1 signaling and the EMT process in CRC cells.** (A) The correlations between the NLRP3 expression and EMT process-related genes, such as Vimentin, ZEB1, ZEB2, SNAI1, SNAI2, and CDH2, within colon cancer tissues from the TCGA database were analyzed with the GEPIA platform. (B) The impact of NLRP3 knockdown on the expression of E-cadherin and Vimentin in CRC cells was assessed using Western blotting. (C) The ImageJ software helped semi-quantitatively analyze the results. (D) Western blotting helped examine the effect of NLRP3 knockdown on p-AKT, p-S6K1, GLI1, and SMO expression. (E) The ImageJ software helped semi-quantitatively analyze the results. NC, negative control, control group; KD, NLRP3-knockdown, NLRP3-knockdown group. ns, no significance, ** *P* < 0.01, *** *P* < 0.001.

Due to the vital link between the proliferative and migratory capacities of tumor cells and the epithelial-mesenchymal transition (EMT) process, our initial investigation evaluated the regulatory function of NLRP3 expression in the EMT progression of CRC cells. We analyzed the correlation between NLRP3 expression and EMT-related genes in colon and rectal cancer tissues from the TCGA database using the GEPIA data analysis platform. The results indicated a linear positive correlation between NLRP3 and Vimentin, ZEB1, ZEB2, SNAI1, SNAI2, and CDH2 expression at the gene level among CRC tissues (Figure 5A, Figure S2). To validate these findings, we conducted Western blotting and observed a significant decline in the expression level of Vimentin in the NLRP3 knockdown group compared to the control group. Meanwhile, there was a notable elevation in the expression level of the E-Cadherin protein (Figures 5B-C).

Previous research has demonstrated that the PI3K-Akt pathway regulates GLI expression, a downstream target of the Hedgehog pathway, via a non-SMO-dependent mechanism involving mTOR. Subsequently, we investigated the potential regulatory role of NLRP3 in the AKT-mTOR-GLI1 pathway. The findings indicated that the phosphorylation of AKT and S6K1 decreased after NLRP3 knockdown and GLI1 expression decreased significantly. However, the expression of SMO, positioned upstream of GLI1, did not demonstrate a significant alteration (Figures 5D-E). We simultaneously down-regulated NLRP3 and overexpressed S6K1. The decrease in the phosphorylation level of S6K1 and the decline in GLI1 expression induced by NLRP3 knockdown were limited by S6K1overexpression. During S6K1 overexpression, NLRP3 knockdown could not cause the decrease of GLI1 expression level (Figures S3A and S3B). Further experiments on proliferation and migration functions produced similar results. Compared to the NC group, the N-KD group exhibited significantly reduced proliferation and migration capabilities. Interestingly, no substantial difference was observed in the proliferation and migration abilities in the SK61 overexpression group (Figures S4A-C). These results suggest that NLRP3 may enhance the AKT-mTOR-GLI1 pathway in CRC cells via a non-SMO-dependent mechanism.

### NLRP3 knockdown inhibited CRC tumorigenesis *in vivo*

**Figure 6:**
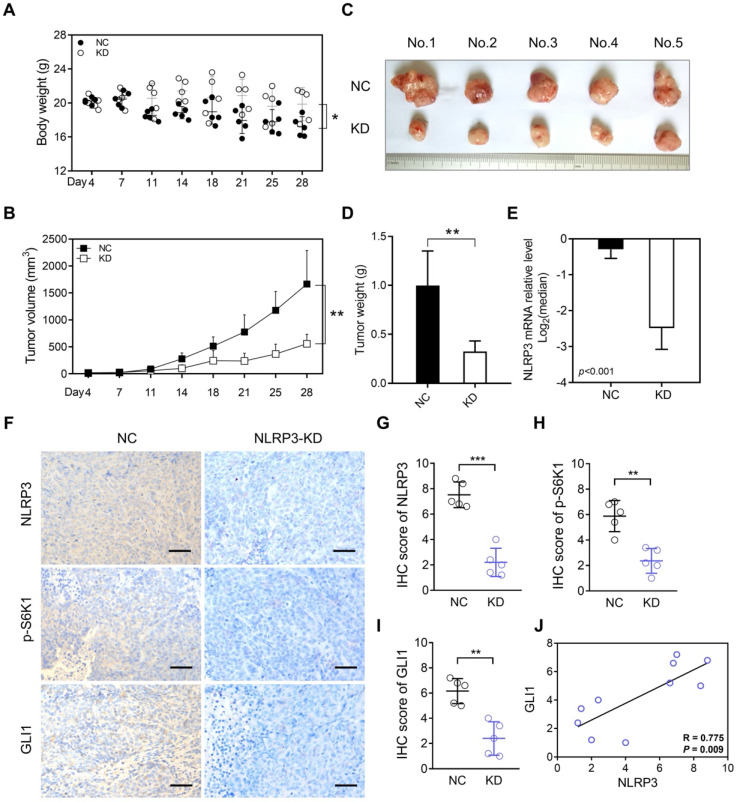
** The inhibition of NLRP3 expression suppresses CRC tumorigenesis *in vivo*.** (A) Weight differences between the NLRP3 knockdown and control groups. (B-D) The differences in subcutaneous tumor volume and weight between the NLRP3 knockout and control groups are shown. (E) Quantifying the expression level of NLRP3 in subcutaneous tumors from both groups using qPCR. (F-I) NLRP3, p-S6K1, and GLI1 expression levels in subcutaneous tumors from mice in both groups were assessed and scored using IHC analysis. (J) A linear correlation between IHC scores of NLRP3 and GLI1 in subcutaneous tumors was established in each respective group. NC, negative control, control group; KD, NLRP3-knockdown, NLRP3-knockdown group. ns, no significance, ** *P* < 0.01, *** *P* < 0.001.

To demonstrate that the abnormally high expression of NLRP3 in tumor cells is a significant factor leading to tumor growth, we conducted subcutaneous tumor formation experiments in mice. Our findings depicted a significantly greater body weight and enhanced nutritional status in the KD group than in the NC group (Figure 6A). Additionally, analyzing the subcutaneous tumor volume and weight indicated suppressed tumor growth in the KD group (Figures 6B-D). Subsequent qPCR analysis of the removed subcutaneous tumors highlighted significantly lower NLRP3 expression in the KD group than within the control group (Figure 6E).

Furthermore, IHC detection and scoring of subcutaneous tumors depicted significantly reduced NLRP3, p-S6K1, and GLI1 expression levels within mice in the NLRP3-KD group (Figures 6F-I). Moreover, correlation analysis indicated a positive association between NLRP3 and GLI1 expression (Figure 6J). Additionally, IHC staining highlighted a significant decline in p-AKT in the NLRP3 knockdown group. However, SMO IHC staining did not significantly differ between the groups (Figures S5A and S5B). These findings suggest that NLRP3 regulates S6K1-GLI1 using AKT, which is upstream of S6K1.

## Discussion

CRC is a prevalent malignant digestive tract tumor characterized by significant morbidity and mortality rates 1, 2, 21. Despite tremendous advancements in pathophysiology, endoscopic or surgical resection, targeted drug therapy, and immunotherapy, the overall survival rate of CRC patients has not improved. However, further exploration is required to better understand the etiology, proliferation, and metastasis mechanisms of CRCs 1, 3. Therefore, it is imperative to investigate the mechanisms involving the proliferation, invasion, and metastasis of CRC to identify effective targets for suppressing tumor progression.

The NLRP3 inflammasome is a cytoplasmic protein complex comprising the NLRP3 receptor, apoptosis-associated speck-like protein (ASC), and a precursor cysteine aspartic protease (pro-Caspase1) 7. NLRP3 is associated with a range of conditions, including systemic inflammation, multiple myeloma, cardiovascular mortality, and malignancy 22, 23. NLRP3 is a pivotal member of the NLR family and regulates intestinal homeostasis while maintaining the immune response 24-28. Our bioinformatics analysis revealed that NLRP3 is significantly overexpressed in CRC tissues. This heightened expression is linked with a less favorable prognosis. Concurrently, our previous pertinent research depicted that NLRP3 was elevated in CRC tissues and related to clinical factors, including lymph node invasion and TNM stage. Moreover, NLRP3-positive patients had a poor prognosis. Univariate and multivariate analysis indicated that NLRP3 expression was an independent prognostic factor involving the survival of CRC patients 20.

Inflammation and chronic infection are associated with the onset of various human cancers 29, 30. Inflammation involves angiogenesis, tumor cell proliferation, and invasion and is pivotal in cancer development 31, 32. Although the involvement of the NLRP3 inflammasome in septic myocarditis has been established, its role and mechanism in malignant tumors remain unexplained 7, 33, 34. To elucidate the regulatory effect of NLRP3 on CRC, NLRP3 knockdown cell lines were generated, which performed gain- and loss-of-function experiments. These findings demonstrated that NLRP3 knockdown effectively inhibited CRC cell proliferation, colony formation, and migration.

EMT is a critical determinant process in tumor metastasis 35-38. Activating the NLRP3 inflammasome can release inflammatory factors, including IL-1β and IL-18, which induce the EMT process, thereby elevating the invasiveness and metastatic capabilities of CRC 39. Analysis with the GEPIA tool of the TCGA database indicated that NLRP3 expression in CRC tissues positively relates to the expression of several EMT-associated markers. Our relevant studies demonstrated that inhibiting NLRP3 expression significantly elevated E-cadherin expression while reducing Vimentin expression in tumor cells, suggesting suppressed EMT progression. These findings provide evidence involving the potential underlying mechanism through which NLRP3 modulates CRC proliferation and migration.

NLRP3 activates tumor-related MAPK signaling pathways, facilitating tumor proliferation and migration during tumorigenesis and development 4, 16, 17. S6K1 is a crucial downstream mTOR signaling cascade mediator critical in CRC onset and advancement. A reciprocal regulatory relationship exists between the mTOR-S6K1 and MAPK signaling pathways 40, 41. Therefore, it is crucial to investigate the regulatory interplay between NLRP3 and S6K1-GLI1 pathways in CRC to decipher the involvement of NLRP3 in tumorigenesis. Our previous research demonstrated a robust correlation between GLI1 and p-S6K1 expression in malignant tumors, particularly among patients with advanced-stage disease. This investigation scrutinized and validated the positive association between NLRP3 and S6K1-GLI1 expression at the mRNA and protein levels within CRC tissues. These findings indicate that the NLRP3-S6K1-GLI1 axis is pivotal in CRC progression.

Emerging research depicts that the PI3K-AKT-mTOR signaling pathway regulates GLI1 expression independently of SMO in numerous cancers, promoting tumor development and progression 42-44. During the study, p-AKT, p-S6K1, and GLI1 expression levels decreased in tumor cells after NLRP3 knockdown. However, no significant difference was observed in the upstream GLI1 target SMO within the Hedgehog signaling pathway. These findings indicate that NLRP3 positively controls the AKT-mTOR-GLI1 pathway via a non-SMO-dependent mechanism in CRC cells.

According to pertinent studies, pyroptosis depends on inflammasome activation, particularly NLRP3, which modulates different physiological functions via two distinct pathways: classical and non-classical. Upon activation, NLRP3 enables the conversion of pro-caspase-1 into its active form. Subsequently, this promotes the production of inflammatory mediators and programmed cell death called pyroptosis. This mechanism can occasionally suppress tumor growth, eliminate damaged cells, and elicit an immune response that could be advantageous for tumor suppression and treatment. Persistent chronic inflammation is a recognized cancer risk factor. Thus, sustained NLRP3 activation could foster an inflammatory microenvironment conducive to tumor progression. Consequently, NLRP3 behaves as a double-edged sword in cancer by mediating pyroptosis since it can either promote or inhibit tumorigenesis 45, 46.

Despite the current research achieving notable progress, several limitations persist. Firstly, although we have explored the cancer-promoting role of NLRP3 in CRC and its potential molecular mechanisms at cellular and animal study levels, our analysis predominantly depends on limited bioinformatics methodologies. Consequently, it is imperative to elevate the sample size of clinical CRC patients for improved data support and gain a more comprehensive understanding. Secondly, the involvement of NLRP3-mediated pyroptosis during the onset and progression of CRC and its upstream regulatory factors remains inadequately investigated 7, 45, 46. Therefore, addressing this will be a crucial focus for future research. Lastly, our subsequent work will examine drug combination strategies and pharmacological profiles from multi-pathway inhibitors to develop innovative methods for advancing precision therapy 47.

Therefore, NLRP3 is implicated in CRC progression and poor prognosis. A positive correlation was observed between NLRP3 expression and the S6K1-GLI1 pathway. The proliferation and migration ability of NLRP3-knockdown CRC cell lines were inhibited. Meanwhile, NLRP3 knockdown effectively suppressed the EMT process. Additionally, p-AKT, p-S6K1, and GLI1 expression levels decreased after the NLRP3 knockdown. However, SMO expression remained relatively unaltered. Finally, S6K1 was overexpressed while NLRP3 was down-regulated. It was discovered that the original NLRP3 knockdown-induced reduction in GLI1 expression was limited due to S6K1 overexpression. This finding suggested that NLRP3 positively controls the AKT-mTOR-GLI1 pathway via an SMO-independent mechanism. Therefore, combined targeted inhibition of NLRP3, S6K1, and GLI1 may represent a novel therapeutic strategy to suppress CRC effectively.

## Supplementary Material

Supplementary figures.
