# Supplementary material for: NLRP3 promotes the proliferation and metastasis of colorectal cancer by regulating the S6K1-GLI1 signaling pathway
Source: J Cancer. 2025 Jan 1;16(2):521–32. doi: 10.7150/jca.101285 (PMC11685681; doi:10.7150/jca.101285)
Supplement: Supplementary file 1 — Supplementary figures. [file jcav16p0521s1.pdf]

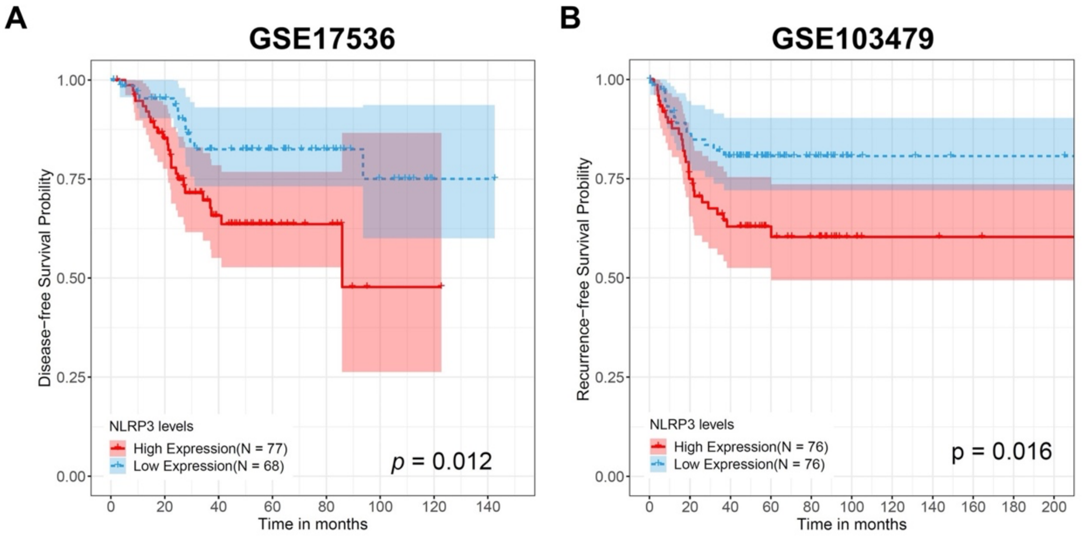

Figure S1. The impact of NLRP3 expression levels on progression-free and recurrence-free survival within CRC patients. (A) The progression-free survival of patients with CRC was evaluated in the dataset (GSE17536) according to NLRP3 expression levels. (B) The recurrence-free survival of CRC patients was assessed in the dataset GSE103479 depending on NLRP3 expression levels.

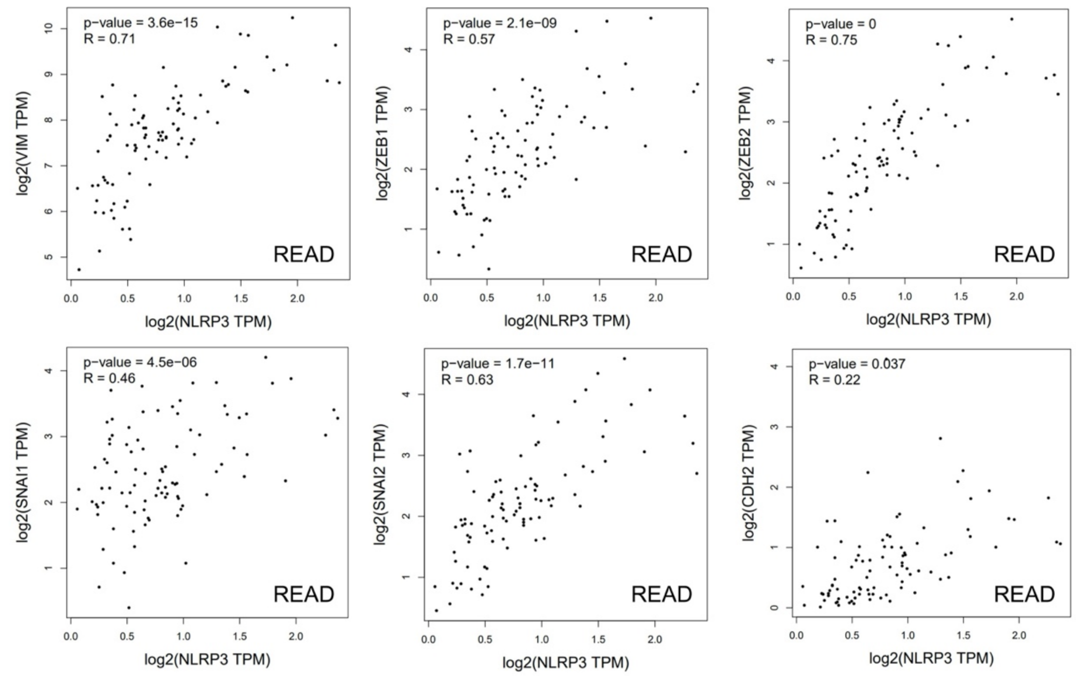

Figure S2. The expression correlation between NLRP3 and EMT process-related genes within rectal cancer tissues. The associations between the expression of NLRP3 and EMT process-related genes, such as Vimentin, ZEB1, ZEB2, SNAI1, SNAI2, and CDH2, in rectal cancer tissues from the TCGA database were analyzed with the GEPIA platform.

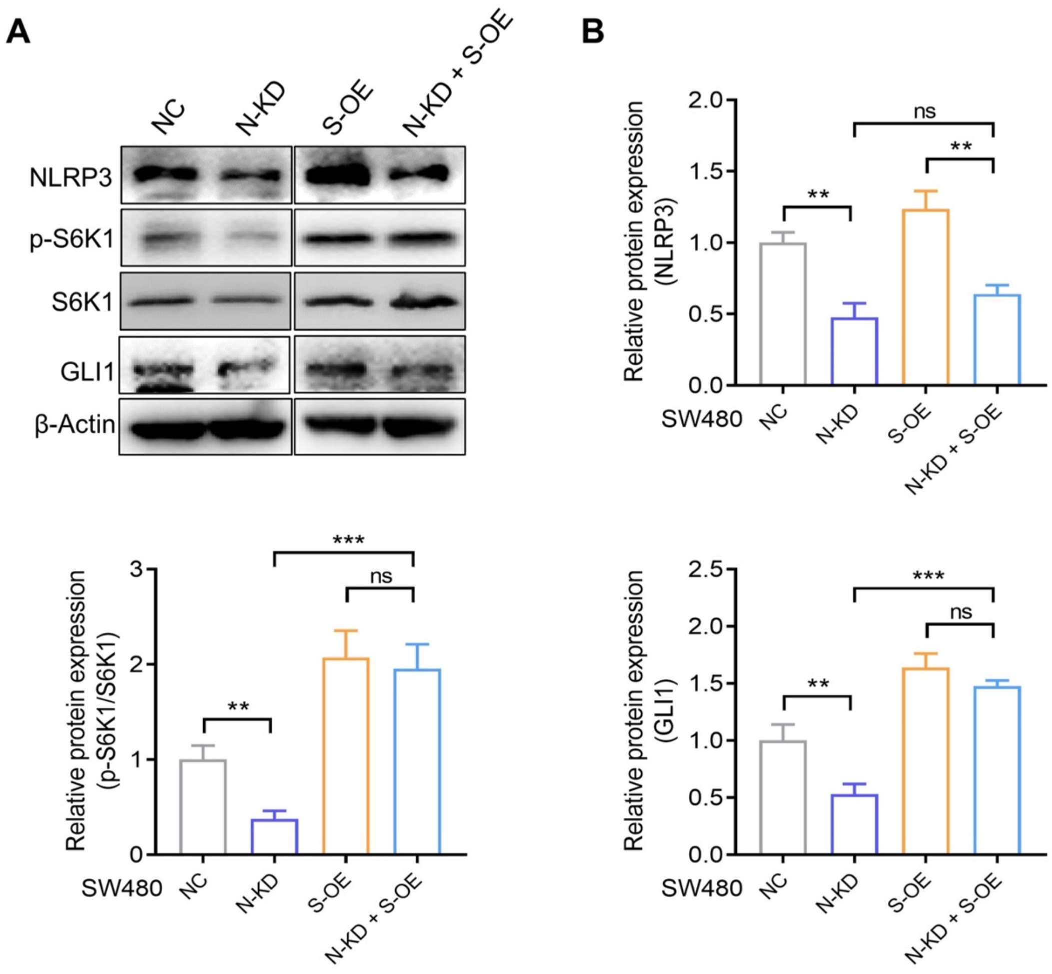

Figure S3. S6K1 facilitates the regulatory impact of NLRP3 on GLI1. (A, B) CRC cells with NLRP3 knockdown and S6K1 overexpression helped assess the expression levels of NLRP3, p-S6K1/S6K1, and GLI1 via Western blotting and semi-quantitatively analyzed using the ImageJ software. NC, Negative Control, control group; N-KD, NLRP3-knockdown, NLRP3 knockdown group; S-OE, S6K1-overexpression, S6K1 overexpression group; N-KD + S-OE, NLRP3 knockdown combined with the S6K1 overexpression group. ns, no significance, ** $P < 0.01$, *** $P < 0.001$.

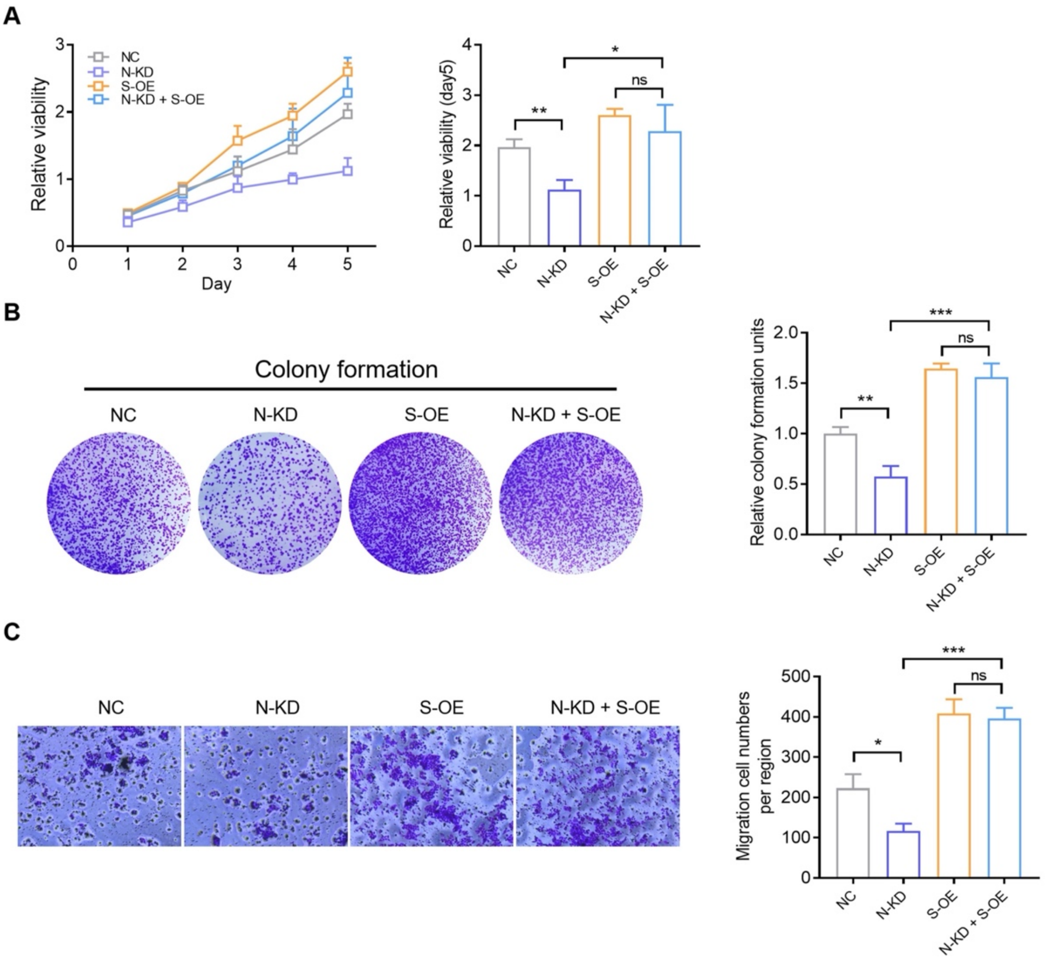

Figure S4. S6K1 overexpression mitigates the inhibitory effects of NLRP3 knockdown on the proliferation and migration of CRC cells. (A, B) The effect of S6K1 overexpression on the inhibitory effect of NLRP3 knockdown on CRC cell proliferation was assessed using the CCK-8 assay and cloning experiments. (C) The Transwell assay helped assess the impact of S6K1 overexpression on the NLRP3 knockdown capacity to inhibit CRC cell migration. NC, Negative Control, control group; N-KD, NLRP3-knockdown, NLRP3 knockdown group; S-OE, S6K1-overexpression, S6K1 overexpression group; N-KD + S-OE, NLRP3 knockdown combined with the S6K1 overexpression group. ns, no significance, * $P < 0.05$, ** $P < 0.01$, *** $P < 0.001$.

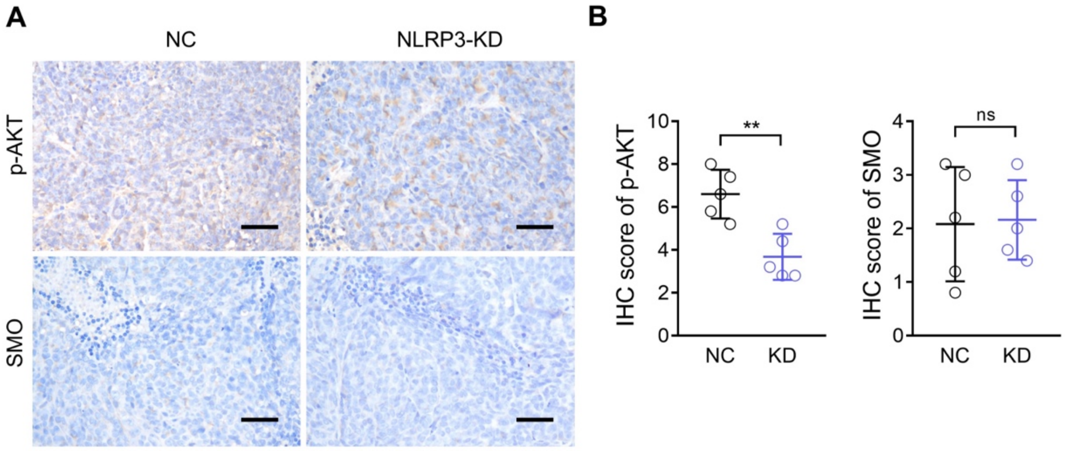

Figure S5. The expression levels of p-AKT and SMO in subcutaneous tumors. p-AKT and SMO expression levels in subcutaneous tumors from mice in both groups were assessed and scored using IHC analysis. NC, negative control, control group; KD, NLRP3-knockdown, NLRP3-knockdown group. ns, no significance, ** $P < 0.01$.
